# Paradigm Changing Integration Technology for the Production of Flexible Electronics by Transferring Structures, Dies and Electrical Components from Rigid to Flexible Substrates

**DOI:** 10.3390/mi14020415

**Published:** 2023-02-10

**Authors:** Franz Selbmann, Soumya Deep Paul, Maulik Satwara, Frank Roscher, Maik Wiemer, Harald Kuhn, Yvonne Joseph

**Affiliations:** 1 Fraunhofer Institute for Electronic Nano Systems ENAS, 09126 Chemnitz, Germany; 2 TU Bergakademie Freiberg, Institute for Electronic and Sensor Materials, 09599 Freiberg, Germany; 3 TU Chemnitz, Center for Microtechnologies, 09126 Chemnitz, Germany

**Keywords:** chip scale packaging, electronics packaging, flexible electronics, integration technologies, metallization, Parylene, transfer, flexible PCB, thin film electronics

## Abstract

Emerging trends like the Internet of Things require an increasing number of different sensors, actuators and electronic devices. To enable new applications, such as wearables and electronic skins, flexible sensor technologies are required. However, established technologies for the fabrication of sensors and actuators, as well as the related packaging, are based on rigid substrates, i.e., silicon wafer substrates and printed circuit boards (PCB). Moreover, most of the flexible substrates investigated until now are not compatible with the aforementioned fabrication technologies on wafers due to their lack of chemical inertness and handling issues. In this presented paper, we demonstrate a conceptually new approach to transfer structures, dies, and electronic components to a flexible substrate by lift-off. The structures to be transferred, including the related electrical contacts and packaging, are fabricated on a rigid carrier substrate, coated with the flexible substrate and finally lifted off from the carrier. The benefits of this approach are the combined advantages of using established semiconductor and microsystem fabrication technologies as well as packaging technologies, such as high precision and miniaturization, as well as a variety of available materials and processes together with those of flexible substrates, such as a geometry adaptivity, lightweight structures and low costs.

## 1. Introduction

Following current trends, such as the “Internet of Things” (IoT), “Industry 4.0”, and “Smart Everything”, the number of sensors to measure non-electrical quantities, such as pressure, temperature, distance, light or concentration of chemical substances, rapidly increases. For example, only the market for microsystems is expected to be 49 billion devices and 22 billion USD, respectively, worldwide by 2027 [1]. 

Driven by wearable applications, the field of flexible electronics gradually became a very relevant topic in recent years. In particular, fully flexible electronics have the benefit of adaption to a surface when mounting on any complex 3D surface or the possibility of following the motions of a body. 

Hence, flexible electronics are currently a key enabling technology for dynamic and flex-to-fit applications in today’s electronic devices. These systems can be used for monitoring the physiological parameters of a person, such as wearable sensors, or for any application that would cause strong stresses when mounting a rigid sensor to any 3D object [2]. Furthermore, flexible electronics are beneficial for the structural health monitoring of lightweight structures due to their low mass and flexibility. 

However, up until now, sensors and actuators are often realized by mechanical microelectromechanical systems (MEMS) using microfabrication technologies on rigid wafer substrates made of silicon, gallium arsenide, or other semiconducting materials. The same applies to microcontrollers based on CMOS technology [3,4,5,6]. Furthermore, the standard technology for the integration of sensors, actuators and microcontrollers into a circuit are rigid printed circuit boards (PCB) based on materials such as FR4. Rigid PCBs combine the advantage of low costs and easy maintenance with good reliability and, hence, are suitable for applications in harsh environments with high mechanical stresses and elevated temperatures [7]. Even though they have a vast range of applications, both of the aforementioned technologies, i.e., the fabrication of sensors and MEMS on rigid wafer substrates as well as their integration on rigid PCBs, are not usable for applications that require thin and flexible substrates [2].

When using flexible substrates, the typical materials are polymers, such as polyethylene terephthalate (PET), polyethylenenaphthalate (PEN), polyimide (PI), polycarbonate (PC), polyethersulphone (PES), polyacrylates (PAR), polycyclic olefin (PCO) and poly(para-xylylene) (Parylene). [8,9,10,11,12,13] In contrast to the other materials, Parylene can be particularly used to realize an ultra-thin and biocompatible flexible PCB with thicknesses of only 10 µm–20 µm. This includes multiple redistribution layers, which are fabricated by using established microsystem technologies and, hence, feature structure sizes as small as 10 µm [11].

With respect to the integration of electrical components, some concepts are established for the direct fabrication of sensors on flexible substrates, e.g., pressure sensors and strain sensors. Usually, this type of sensor directly uses properties of the substrate itself as the strain sensor bases on the stretchability of the substrate or requires the usage of new materials [14,15,16]. Printing technologies can be directly performed on flexible substrates for the realization of electrical interconnects and contacting chips and the fabrication of sensor structures themselves. Hence, by printing technologies, devices such as pressure sensors [17], radio frequency identification tags (RFID) [18,19], solar cells [20], light emitting diodes (LED) [21], transistors [22], passive microwave antennas [23] and electrical chip interconnects [24] can be realized. However, the variety of devices and the performance that this technology can realize are limited. Moreover, in general, the direct processing on flexible substrates is limited since the established substrate materials are neither compatible with the standard wafer-level processing using aggressive chemicals and high temperatures nor do the non-wafer-level methods for the direct processing on flexible substrates, such as additive manufacturing, offer a sufficient resolution for the fabrication of complex and highly integrated devices. Furthermore, most of the established standard packaging methods, such as soldering or bonding, can only be applied to flexible substrates, but with limitations due to inappropriate process conditions, particularly high temperatures. Particularly, the application of standard packaging and integration methods is not possible for the established ultra-thin Parylene flexible PCB [11]. 

In order to combine the advantages of ultra-thin, flexible substrates and electronics with those of conventional micro technologies, a new approach for the fabrication of flexible electronics is the transfer of structures and components fabricated on rigid wafers using conventional technologies and a subsequent transfer to a flexible substrate. Kim et al. presented an interesting approach for the transfer of graphene from rigid to flexible substrates. Considering the high temperatures above 1000 °C needed for the fabrication of graphene, established materials for flexible substrates cannot be used for direct deposition. Hence, the authors deposited graphene on a copper substrate and deposited Parylene C layers of 15 µm and 25 µm, followed by peel-off, the electrochemical delamination of Parylene or the etching removal of the substrate. In doing so, the graphene was pulled off together with the Parylene and, thus, was transferred onto a flexible free-standing Parylene substrate. [25] Similarly to this, Kim et al. demonstrated the transfer of inkjet-printed silver electrodes to Parylene using peel-off from a PDMS sacrificial layer [26].

The requirement for the success of the peeling-off method is an insufficient adhesion of the material to be transferred to the rigid carrier substrate and good adhesion to the Parylene. In order to apply this method to multiple material systems and transferred structures of higher dimensions rather than a single atomic layer, we propose the usage of an additional sacrificial layer.

In this study, we investigate the adaption and extension of the described transfer process to transfer the metallic structures deposited by different technologies, as well as whole dies, electronic components and multiple materials systems, to an ultra-thin, flexible Parylene C substrate. Parylene C (Poly(2-chloro-p-xylylene)) is used due to its outstanding combination of excellent properties, which are as follows: a highly 3D conformal deposition from the gas phase and at room temperature, low Young’s modulus (flexibility), biostability and biocompatibility, optical transparency, chemical inertness, dielectric properties and low permeability for gases and moisture [27,28,29].

**Figure 1:**
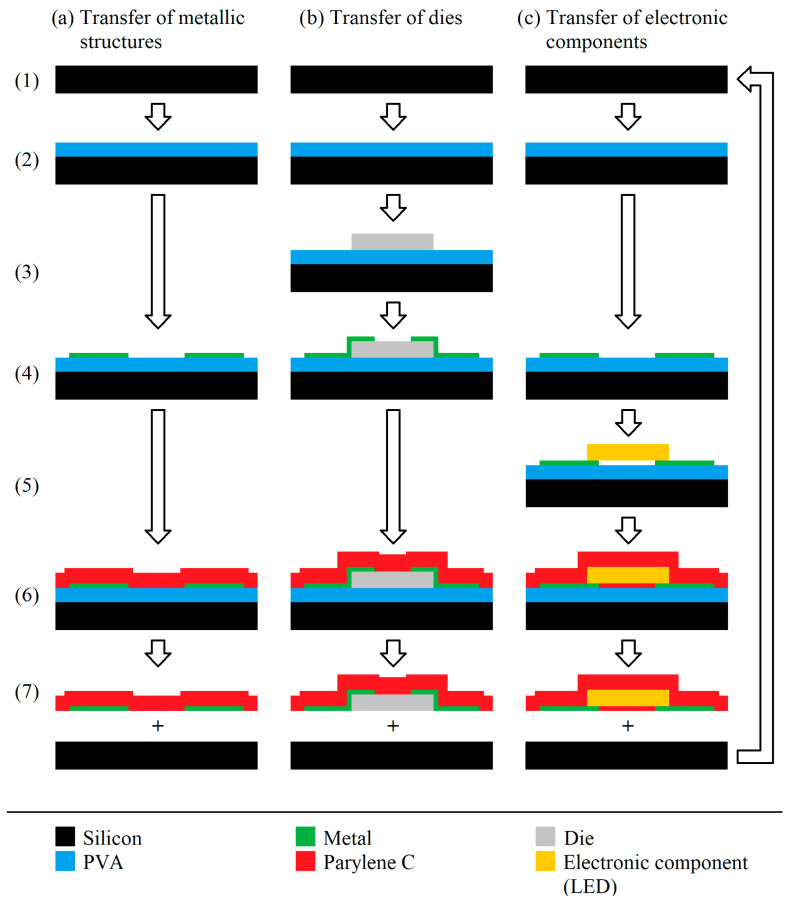
Process flow for the transfer of (**a**) metallic structures, (**b**) semiconductor dies, (**c**) packaged electronic components from rigid carrier substrates (1) to free-standing Parylene, including the sub-process of sacrificial layer deposition (2), die attachment (3), metallization (4), integration of electronic components (5), Parylene coating (6) and lift-off (7).

The approach for the transfer process of structures from rigid substrates to flexible free-standing Parylene substrates used in this paper is depicted in Figure 1. In doing so, different scenarios for the transfer of (a) metallic structures, (b) semiconductor dies, and (c) packaged electronic components are differentiated.

**Figure 2:**
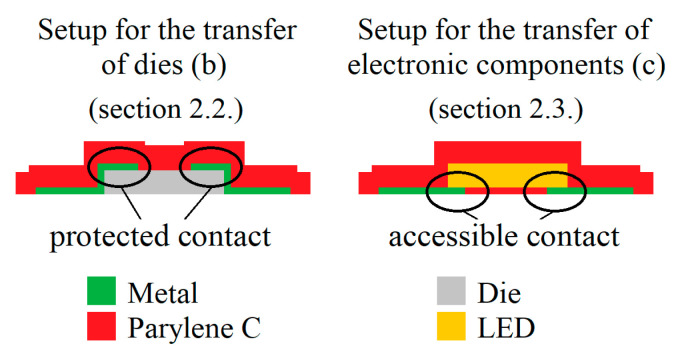
Difference in test setup for the transfer of dies (b) and electronic components (c) with Parylene-protected contact areas and accessible contact areas, respectively.

It is important to note the differences in the setup for the transfer of the dies and electronic components (Figure 1b,c). Whereas the electrical contacts between the silicon die and the printed parts are covered and protected with Parylene in the final setup for (b), these contacts are accessible for the setup described for (c). The difference is schematically depicted in Figure 2 with the related sections in this paper.

## 2. Materials and Methods

**Figure 3:**
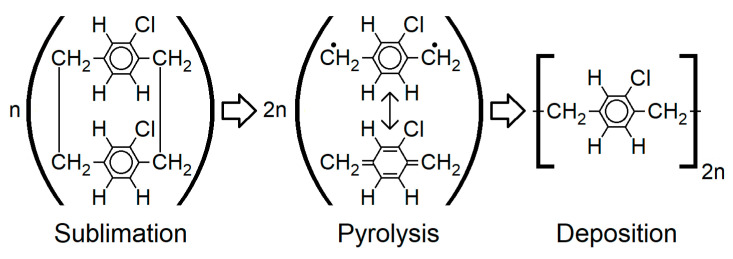
Molecular structure for each step of the Gorham CVD process for the deposition of Parylene [27].

For all the experiments, 6″ silicon wafers or silicon chips, respectively, were used as rigid carrier substrates (Figure 1 (1)). For the sacrificial layer, 80% hydrolyzed polyvinyl alcohol (PVA) was used, which was obtained from Sigma Aldrich. The PVA was spin-coated at 1500 rpm for 20 s in an aqueous solution of 4.7 w%, followed by subsequent drying at 105 °C for 5 min in air (2). The PVA was chosen as a sacrificial layer since it can be dissolved by water, and thus, a completely solvent-free process can be realized. After the deposition of the sacrificial layer, the structure to be transferred is deposited (3) to (5), whereas different scenarios were tested, including (a) the transfer of metals only, (b) the transfer of semiconductor dies with subsequent metallization, and (c) the transfer of packaged electronic components with previous metallization, respectively. Subsequent to this, 15 µm of Parylene C was deposited by chemical vapor deposition (CVD) using the Gorham process, as depicted in Figure 3, and a Plasma Parylene LC 300 RW from Plasma Parylene Systems GmbH (PPS), Germany (6). In doing so, in the first step, 30 g of a solid dimer was sublimed at 130 °C, followed by pyrolysis at 740 °C, and hence, thermal cracking into monomers. In the final step, the linear polymer chains were formed at 40 °C. The setup was completed by a cool trap at −80 °C and a vacuum pump [27,28,29].

For lift-off, a scalpel was used to cut the Parylene film at the edge and provide access for the water. Afterwards, the substrate was placed in DI water at room temperature overnight to dissolve the PVA and release the Parylene, including the transferred structures (7).

### 2.1. Transfer of Metallic Structures

**Figure 4:**
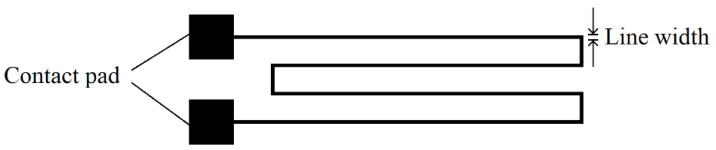
Schematic design of the metallic test structures (not to scale).

For the transfer of metallic structures, different metals and deposition methods were investigated following the general process flow depicted in column (a) of Figure 1. For all metallic structures, the meander design given in Figure 4, with a total length (l) of ~11 cm, was used. The line width (w) was varied between 100 µm and 250 µm. For the investigation of the transferability of larger area structures, a metallic pad of 2 mm × 2 mm was realized on each end of the meander. In order to verify the function of the structures after the transfer, these pads were connected, and electrical measurements were performed.

The metallic structures were realized by the maskless direct deposition of nanoparticle silver inks using aerosol jet printing (AJ300 system of Optomec Inc., USA) and subsequent sintering post-treatment. In order to compare the influence of the material and ink composition on the transfer results, two silver nanoparticle inks were used: Bando SW1020 on the water base and GenesInk S-CS31506 on the solvent base. Both inks are based on silver nanoparticles and were used as received. The printing settings for printing with the Bando Ink were 800 sccm for the atomizer, 750 sccm for the exhaust, and 60 sccm for the sheath gas. The substrate temperature was maintained at 80 °C, and the printing speed was kept constant at 8 mm/s. When printing with GenesInk, the atomizer, exhaust and sheath gas flows were set at 950 sccm, 925 sccm and 60 sccm, respectively, whereas the printing speed was 5 mm/s. The sintering condition was 120 °C in air for 2 h for both inks. In order to investigate the influence of the height of the structure to be transferred, a second group of meanders was printed twice.

Alternatively to the printing, the metal structures were realized by sputtering. For this, shadow masks with the same design given in Figure 4 were fabricated by laser ablation in sheets of stainless steel. The line width in the shadow masks varied between 75 µm and 100 µm. Gold and platinum were sputtered on PVA-coated silicon chips using a desktop sputter tool (BalTec SCD 050 Sputter Coater). The sputtering was performed at a pressure of 0.04 mbar and a current of 60 mA. In order to vary the thickness of the sputtered structures, the two metals were deposited with different sputtering times of 400 s and 750 s each. After sputtering, the Parylene was deposited, followed by a subsequent lift-off. 

The thickness of all the metallic structures was measured before the deposition of Parylene C using a KLA Tencor Alpha-Step 500 Surface Profilometer and averaged over three different measurement positions. After the deposition of Parylene C, the lift-off of the structures was performed, and the electrical resistance (R) was measured to compare its values before the lift-off. In order to prove the success of the transfer process, the electrical resistance of each metallic structure, as well as of the transferred chips, was measured before and after the transfer process using a PeakTech 2010DMM multimeter. Afterwards, the electrical conductivity (σ) was calculated by the following equation, considering the height (h) and width (w) of the metallic structures as well as their total length (l) of 11 cm.

(1) $$\sigma \;=\frac{1}{R}\;\cdot \;\frac{l}{w\cdot h}$$

Additionally, the samples were bent on some cylindrical test specimens with different diameters (d). The used diameters are given in Table 1, as well as the strains (ε), which are introduced by the bending test. For the calculation of the strain, the following equation was used, assuming a sample thickness (t) of 15 µm.

(2) $$\varepsilon =\frac{t}{d+t}\cdot 100\;\%$$

During the bending test, the samples were observed concerning delamination for diameters of ≥1 cm, and their electrical resistance was measured to calculate the electrical conductivity, as described above.

### 2.2. Transfer of Dies

In order to investigate the integration of whole dies and chips, respectively, the process flow depicted in column (b) of Figure 1 was followed. Semiconductor dies were thinned to achieve some flexibility using the BrewerBond^®^ process described elsewhere [30]. It can be briefly summarized as follows: spin coating of an adhesive on a carrier wafer as well as the wafer to be thinned, followed by subsequent temporary bonding and wafer thinning by grinding and chemical mechanical polishing. Afterwards, the temporarily bonded wafer compound is attached to a dicing frame, and the carrier wafer is mechanically debonded, followed by dicing. Additionally, these chips were metallized before positioning the carrier substrate by sputtering gold and platinum for 750 s and 1000 s using the same tool and parameters as mentioned earlier. Both metals were selected due to their different adhesion on silicon: gold adheres poorly to silicon without any additional adhesion-promoting layer, whereas platinum adheres well. The thinned and metallized 5 mm × 5 mm silicon chips of 60 µm thickness were positioned on the PVA by pick-and-place. In order to achieve good sticking, the chips were positioned while the PVA was still liquid. It is important to note that the metallized and, thus, conductive side of the chip was covered by Parylene after the transfer (Figure 2, left setup). Hence, gaining direct access to enable electrical contacting is not possible. 

For transferring the devices with top-side contacts that are covered and protected by Parylene after the transfer process, it is important to investigate how the external access can be realized for the electrical contacts without patterning and opening the Parylene itself, respectively. For this reason, conductive paths were printed on the metallized side of the chip, and the contact pads were printed on the PVA as well as the connection of these contact paths with the contact pads. For the printing, the aerosol jet and GenesInk S-CS31506 were used again, with the parameters described in Section 2.1. Since the conductive path between the contacts on the chip and the pads on the PVA has to bridge the step induced by the chip thickness, all structures were printed twice. After printing the contact pads, Parylene deposition and lift-off were performed as described in Section 2.1.

### 2.3. Transfer of Electrical Components

In addition to the transfer of silicon dies, the transferability of electronic components is also of practical relevance (see column (c) in Figure 1 as well as Figure 2). Hence, bulky packaged LEDs of 2.0 mm × 2.0 mm × 1.4 mm width, length and depth, which are usually designed for surface mounting (SMD), were used to investigate the transferability. The LEDs are obtained from CML innovative Technologies (CMD28-21 Series SMT LEDs) and have a red color, a maximum forward voltage of 2.8 V and a current of 20 mA.

For the sample preparation, two contact pads and two electrical connectors were printed on the PVA using the aerosol jet and the GenesInk S-CS31506 again, as described in Section 2.1. Afterwards, the LED was glued onto the printed electrodes using conductive glue LOCTITE ABLESTIK ICP 4015, which is a silver and silicone-based glue. The glue was cured in accordance with the datasheet at 80 °C for 30 min in air. After this, the Parylene deposition and lift-off were performed. In order to prove the successful transfer of the LED, it was tested as to whether it was still glowing after the transfer when applying a forward voltage using a Statron power supply, type 3225. 

### 2.4. Transfer Mechanism

**Figure A1:**
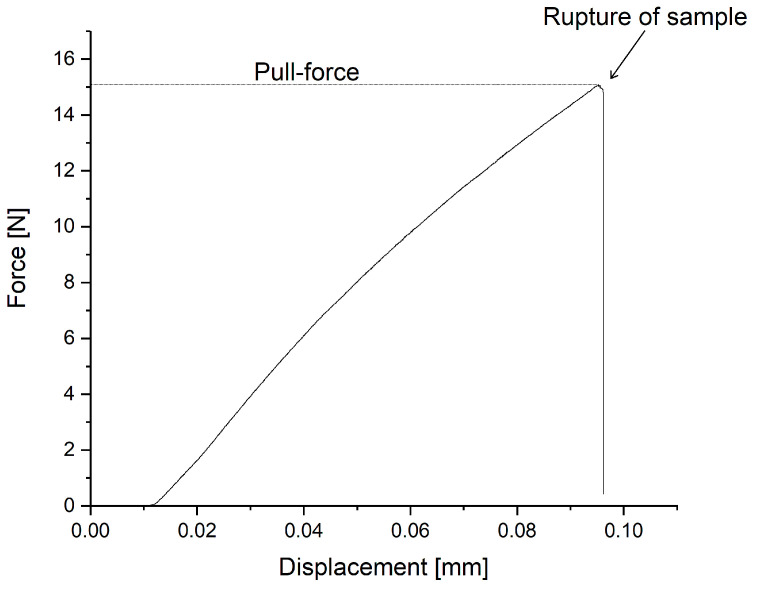
Example of the measurement results obtained by the tensile test and the deriving of the pull-force.

For a better understanding of the transfer mechanism, the adhesion of the metallic structures was tested in several ways. First, all flexible metallic structures were tested by a tape test in similarity to ASTM D3359. In doing so, the samples were glued with their back side (the side without any metallic structures) to silicon wafers with double-sided adhesive tape (TESA 4965). In the next step, another adhesive tape (TESA 4289) was attached to the front side and slowly peeled off again after a waiting time of 1 min. Afterwards, the samples were inspected. The adhesive tapes used for the test were selected to use the tape with stronger adhesion (11.8 N/cm on stainless steel) for the fixation of the sample on the silicon and the one with less adhesion (5.5 N/cm on stainless steel) for the peel-off. For reference, the described tape test was also performed for all metals, which were deposited directly on Parylene. In order to quantify the adhesion strength of the transferred metal on Parylene, pads with a size of 1 cm × 1 cm were printed using the described aerosol jet technology and Genesink S-CS31506 and transferred onto Parylene. For comparison, the Parylene was metallized directly with the same technology. Both samples were metallized with 12 pads, lifted, and the pads were cut using regular scissors. Next, two stainless steel studs with a contact area of 5 mm × 5 mm were glued onto the metallic pad and Parylene, respectively, using Pattex “Sekundenkleber” (liquid cyanoacrylate glue). The glue was dried overnight in ambient conditions. For the measurement of the tensile strength, a TIRA Test 2805 was used with a force sensor (serial number 63026, K series) with a nominal load of 1 kN and a sensitivity of 2 mV/V. Within the tensile test, the pulling force was measured based on time and displacement, and the pull force was derived from its peak value (Figure A1 in Appendix A). In doing so, a testing speed of 3 µm/s and a pre-force of 5 N were applied. The two surfaces of the ruptured sample on the studs were analyzed in the light microscope (Eclipse L200, Nikon Corporation, Japan) to determine the rupture area and calculate the adhesion strength of the metal to the Parylene. Finally, this experiment was repeated using the same equipment and procedure but an additional high-speed video capture of the rupture event.

**Figure A2:**
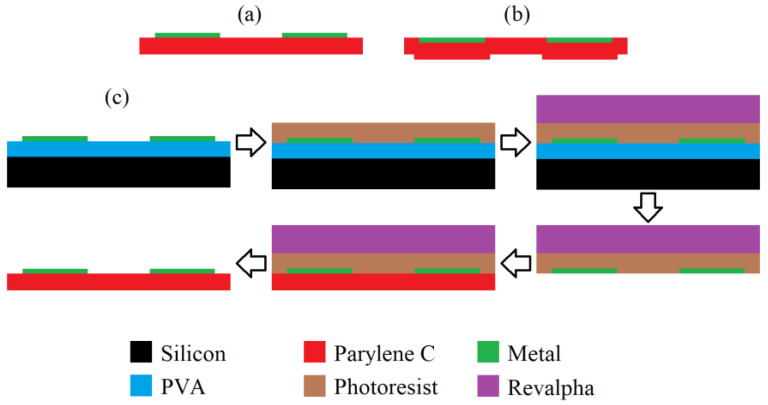
Metallic structures on Parylene obtained by direct metallization (**a**) and the transfer process (**b**). Process for the realization of a transferred metallization that features only one interface with Parylene (**c**).

As indicated in Figure 1 (7) and Figure A2 in Appendix B, respectively, in contrast to the conventionally metallized Parylene samples, the transferred structures were embedded into the Parylene and had interfaces on three sides, i.e., on the top side and the edges. In order to analyze whether the additional interfaces of Parylene with the transferred structures influence the adhesion compared to Parylene, which was metallized directly, a sample was prepared in which the transferred metal structure features only one interface with Parylene. The process is described in detail in Appendix B. Afterwards, the described tape test was performed on the obtained metallized Parylene. For a detailed analysis of the interfaces between the transferred structures and Parylene, the samples were embedded into epoxy (EpoFix Harz and a related hardener by Struers GmbH, Germany), outgassed in vacuum and hardened for 24 h. Subsequently, a cross-section was made using silicon carbide-based abrasive paper, as well as MD-Dur and MD-Nap polishing wheels with a DiaPro diamond suspension (3 µm and 0.25 µm, respectively). After fabrication, these cross-sections were analyzed by light microscopy.

Finally, the roughness of the metallic structures was investigated before and after the transfer process. In doing so, the roughness of the conductive path was measured using confocal microscopy (confovis GmbH, Germany) after steps (3) and (6) of the process depicted in Figure 1a and on the accessible metal side. Since the aerosol jet-printed structures (GenesInk S-CS31506) feature the highest roughness, this metallization method was selected for this investigation.

## 3. Results 

### 3.1. Transfer of Metallic Structures

**Figure 5:**
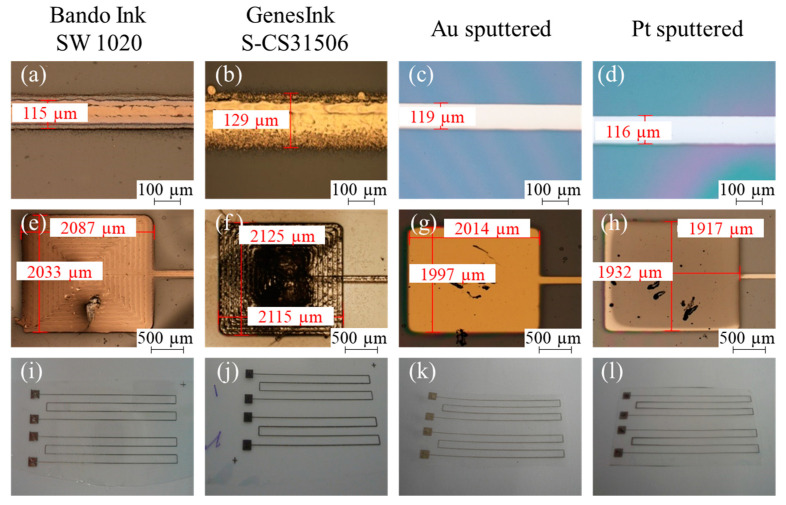
Representative front side light microscopic images of obtained lines (**a**–**d**) and pads (**e**–**h**) before the transfer, as well as photos of transfer results (**i**–**l**) for the different materials and deposition technologies, respectively.

The deposition results for the different metals and different deposition technologies, respectively, are summarized in Figure 5 and Table 2, together with the results for the measured electrical conductivities. For the printed structures, the Bando Ink shows a higher line width and thickness when printed twice. However, in contrast to the GenesInk, Bando Ink shows cracks after the sintering process and is transferred only partially for some samples. A potential reason for this different behavior could be the different chemical compositions of the inks or different particle sizes. Due to the crack formation and only partial transfer for the Bando Ink, for all the following experiments, GenesInk is used. In comparison with Bando Ink, GenesInk features a smaller line thickness and a similar line width when printed once. Furthermore, aerosol jet-printed meanders show a sintered structure with a high roughness, whereas the sputtered meanders show a very smooth surface. For the sputtered metal structures, the line width is slightly larger than the targeted mask width, which is related to some deviations during the laser fabrication of the shadow masks. The sputtering time correlates with the obtained metal thickness. However, using the same parameter set, the obtained platinum structures are thinner than the gold structures. Independent of the material and metallization technology, the pad size was approximately 2 mm × 2 mm.

Independent of the metals (silver, gold and platinum) and the deposition technology, all the structures are transferred successfully from the rigid substrate to the flexible one. The success of the transfer process for the metallic structures is indicated by the electrical conductivity of the transferred structures, measured before and after the transfer. However, for the transfer result of the printed metal structures, a dependency on the used ink is noticeable. Here, a more detailed study is required to investigate the impact of the chemical composition of the ink, i.e., solvent vs. water bases and pure metallic vs. composite particles, as well as the impact of the particle size and shape, i.e., spherical particles vs. nanowires [12,13] on the transfer result.

In particular, the transfer works successfully for thin structures of only some tens or hundreds of nm as well as for thicker ones of several µm. It is interesting to note that even the hand-written labels on the sample were transferred, as can be seen in Figure 5j. Hence, it can be concluded that the transfer process also works for organic materials. Furthermore, the lateral dimensions do not influence the transfer results; the thin metal lines with a width in the µm range are transferred as well as the larger pads with dimensions in the mm range. The change in the electrical conductivities before and after the transfer process is very small and proves that the structures remain intact and functional during the transfer. Note that the absolute values for the electrical conductivities also include the other features of the test structures, such as the contact resistances, etc., and hence, cannot be directly compared to the values in the scientific literature. The mismatch of the absolute electrical conductivities of the different gold samples is most likely caused by inaccuracies in the layer thickness measurements. 

It is interesting to note that the electrical conductivity of the transferred structures per tendency has slightly higher values than before the transfer process. The only exception is the sputtered (750 s, 75 µm mask width) platinum sample. However, this sample received some damage during the electrical measurement and, hence, is not representative. Potential reasons for the slightly increased conductivities after the transfer process are some residues and oxides on the front side (the sample side, which is accessible before the transfer), whereas they have a clean back side (the side which is accessible after the transfer). However, the used metals are noble metals with an unlikely oxide formation; though, particularly gold and platinum show an increase in conductivity. Hence, this explanation is not very likely. Another possibility is a different surface roughness on the two sides, in particular for the printed structures. This approach is investigated in detail in Section 2.4 and Section 3.4. Finally, a third possibility is that the contact probe for the electrical measurement has better contact when the sample is on a flexible and soft substrate since it can deform or slightly penetrate into the structure, compared to a measurement on a rigid substrate.

**Figure 6:**
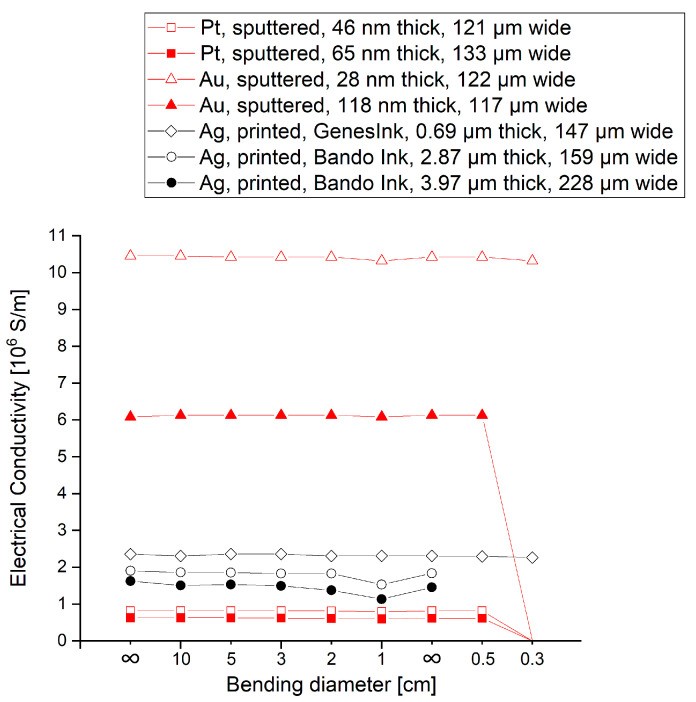
Electrical conductivities of the transferred metal meanders for different bending diameters.

Figure 6 depicts the results of the bending tests, i.e., the electrical conductivity of the metallic meander structures at different bending diameters. Note that the bending diameter was decreased stepwise from 10 cm to 1 cm; however, before bending at a smaller diameter, the samples were flattened again to investigate the reversibility.

The initial values for the different metals, thicknesses and deposition technologies are in accordance with the theory: By comparing the same material and technology, thicker samples always feature a lower electrical resistance than thinner ones. The sputtered gold structures feature a higher electrical conductivity than the sputtered platinum structures due to the lower resistivity of the material gold itself, and the printed structures feature a lower electrical conductivity compared to the sputtered gold structures due to their sinter structure. 

The sputtered gold meanders show no dependency on the bending diameter; however, the thick gold structures are destroyed when bending below a 0.5 cm diameter. Similarly to this, the platinum structures show no dependency on the bending diameter either and are also destroyed when bending diameters below 0.5 cm are applied. All printed structures show a decrease in electrical conductivity with decreasing bending diameters; however, this dependency is stronger for the Bando Ink than for GenesInk, comparing, e.g., the values at a 1 cm bending radius. However, even this stronger decrease in electrical conductivity is reversible for the Bando Ink. Furthermore, it can be noted that the structures printed by GenesInk are not destroyed at smaller bending diameters. The results prove that the transferred metal structures can be bent reversibly to bending diameters of at least 1 cm without any damage, such as delamination. 

### 3.2. Transfer of Dies

**Figure 7:**
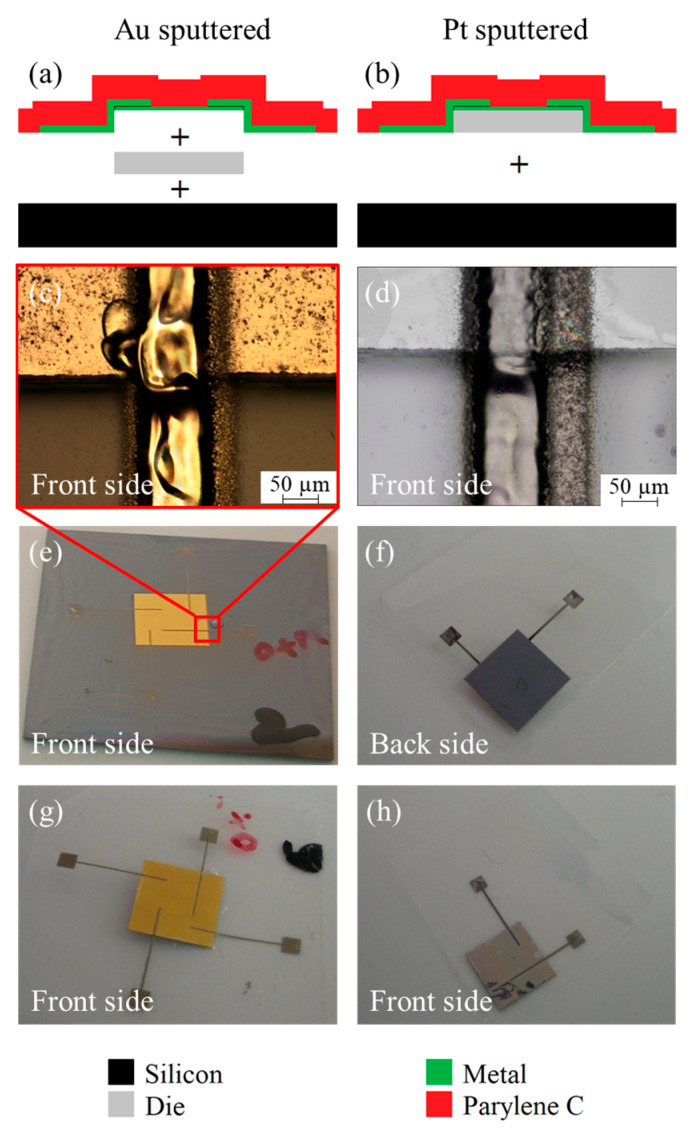
Schematics of the transfer results for a die with gold (**a**) and platinum (**b**) metallization as well as photos and light microscopic images of the silicon chip with printed contacts before transfer (**c**–**e**) and the transferred structures (**f**–**h**), whereas (**f**,**h**) show the front side and back side, respectively.

The transfer result of the die, including the printed contacts, depends on the metal layer: For platinum with good adhesion to silicon, the silicon die, including the platinum metallization and the printing, is transferred to the Parylene. However, for gold with low adhesion to silicon, the silicon and the metallization layer of the die are separated during the transfer process. Hence, only the metallization layer, including the printed contacts, is transferred to Parylene, as depicted in Figure 7.

This dependency of the transfer result on the metallization layer opens a tunability; if the whole die, e.g., due to the embedded electrical components, needs to be transferred, a metallization layer with high adhesion is required. In contrast to this, only metal structures can be transferred when metals with low adhesion are used. This opens up a high process flexibility since metallized chips from different sources can be positioned by pick-and-place, followed by subsequent contacting. In particular, by using this approach, it is also possible to transfer small structures that are realized by conventional microtechnology.

For the platinum metallized die, the electrical resistance was measured before and after the transfer process. The results are summarized in Table 3 and prove that the electrical contacts are still functional after the transfer process, even though their electrical resistance was increased by approximately one order of magnitude. This increase is assumed to be caused by the fact that the conductive path was printed over the edge of the die with a thickness of 60 µm, resulting in a lower thickness of the printed structure at exactly the edge of the chips (Figure 7c,d). Hence, the conductive paths feature lower mechanical stability at this position and are easily subject to mechanically induced damages. Probably, this effect can be reduced by using dies with a trapezoidal cross-section.

Nevertheless, depending on the metallic material, the described method can transfer whole dies (including electrical and sensing devices together) with their electrical contacts to flexible substrates.

### 3.3. Transfer of Electrical Components

**Figure 8:**
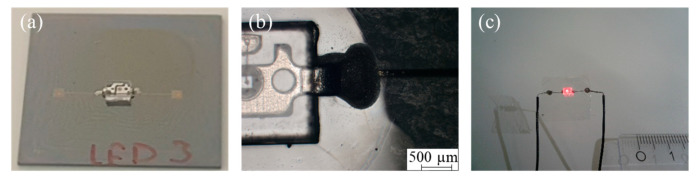
Photo of the silicon chip with printed contacts and mounted LED before Parylene deposition (**a**) as well as front side light microscopic image of the LED contacts (**b**). Photo of the lifted LED on the flexible Parylene substrate when applying a voltage (**c**). The setup corresponds to Figure 1 (column (c) and line (7)).

The bulky surface-mounted LED is transferred to the flexible Parylene successfully as an example of the transfer of electrical components and their integration to a flexible Parylene substrate according to the process scheme in Figure 1c. Figure 8 depicts the detailed setup before (a,b) and after the transfer process (c). After applying the DC voltage to the wires glued onto the printed contact pads, the LED starts to glow, as can be seen in Figure 8c. In conclusion, the whole setup comprising the printed conductive paths and contact pads, as well as the LED, can be transferred to the flexible Parylene substrate without any damage or loss of electrical contact. Again, it is worth noting the difference between the results in Section 3.2.: In contrast to Section 3.2., where the contacts of the transferred chips are finally protected by Parylene and are electrically inaccessible, in this section, the setup is realized in a way that all the contacts are unprotected and still accessible after the transfer.

### 3.4. Transfer Mechanism

The results of the tape test for the investigation of the adhesion of the metals on the Parylene are summarized in Table 4 for the direct metallization of the Parylene and for the metallization by the transfer process, both without any further surface treatment.

As indicated in Table 4, the metallization by the transfer process per tendency shows an improved adhesion when compared to the direct metallization. This effect is particularly obvious for sputtered gold. Gold, which is directly sputtered on Parylene, shows almost no adhesion and hence, thin adhesion-promoting layers of titanium or chromium are commonly used. Therefore, the transfer process is not only a new option for the metallization of Parylene, but it even simplifies the process since no additional adhesion-promoting layers are required. The difference between Bando Ink and GenesInk could be caused by the crack formation in Bando Ink. For some samples with transferred metallization, the Parylene was destroyed during the test; however, the metal was not separated from the Parylene. 

**Figure 9:**
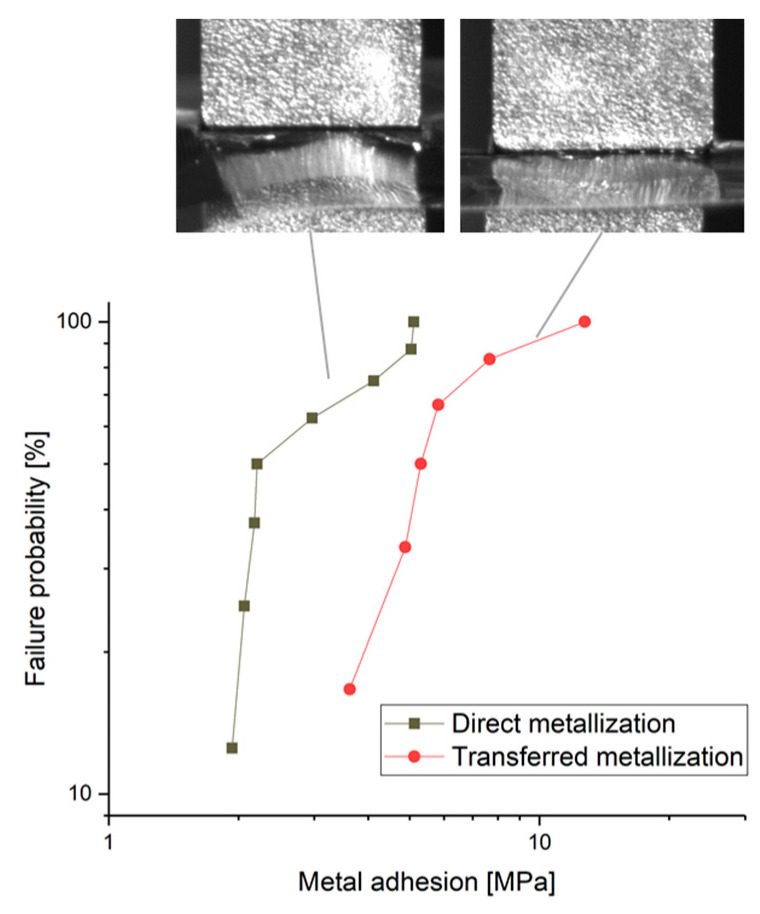
Weibull plot of the tensile strengths for GenesInk on Parylene, applied by direct metallization and by the transfer process, respectively. The inset shows photos captured by a high-speed camera during the tensile test at the moment before rupture. Note that the upper stud is mirrored in the metallization of the sample and the Parylene foil.

For the quantitative analysis of this effect, the tensile test was performed using GenesInk, which was printed directly or transferred, respectively. The results are summarized in the Weibull plot in Figure 9. Note that not all the twelve samples of each group were considered for the diagram since, for some samples, the glue for the attachment of the stud failed, or the Parylene itself was ruptured. In both cases, the obtained values are not significant for the adhesion. Figure 9 indicates that the transferred GenesInk shows an increased adhesion compared to the directly printed metal by a factor of around 2.0–2.5. This result is in accordance with the qualitative tape test and Table 4.

Furthermore, the Weibull plot in Figure 9 suggests different failure mechanisms, indicated by the different slopes of the graphs. For direct metallization, three different modes cause the loss of adhesion. The transferred metals show only two different modes. The photos captured by the high-speed camera during the tensile test are given in the respective inset of Figure 9. These photos support the indication of the Weibull plot of different failure modes: the image for the direct metallization shows a narrowing before the rupture, and the image for the transferred metallization suggests a holohedral rupture, which is also obvious when inspecting the ruptured samples. Nevertheless, a more detailed analysis is required for the investigation of the failure mechanisms in the future, which is not the focus of this study.

In order to study whether the improved adhesion for the transferred metals is caused by the additional and increased contact surface of the metal with the Parylene (see Figure A2), an additional transfer step was performed, as described in Section 2.4 and Appendix B, respectively. In doing so, a sample with transferred GenesInk but a reduced metal-Parylene contact surface, similar to the one obtained by direct metallization, is prepared. The tape test reveals no difference in adhesion between the transferred samples. Both samples, transferred once or twice, show excellent adhesion. Hence, it can be concluded that the adhesion does not depend significantly on the contact surface of the metal to the Parylene. Another reason for the increased adhesion of the transferred metals to the Parylene could be that there is a different chemical bonding since the Parylene is not exposed to air before coming into contact with the metal. However, additional investigations are required in the future as well to explore the reason for the improved adhesion.

**Figure 10:**
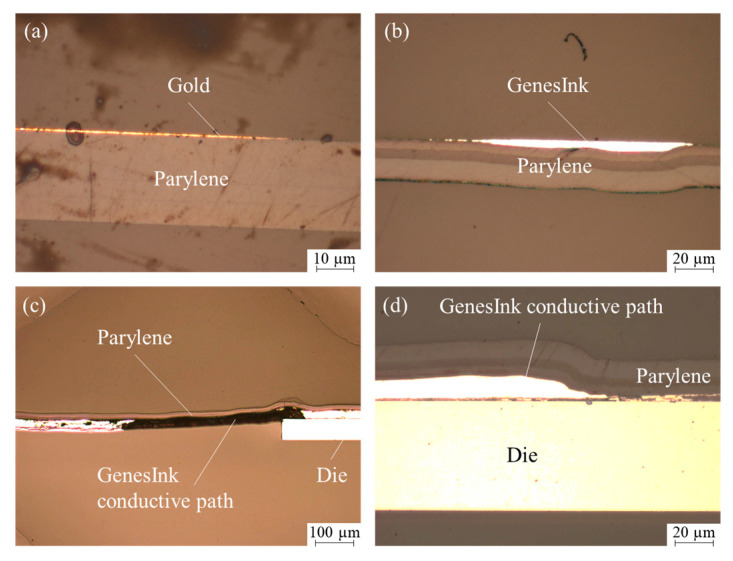
Light microscopic images of cross-sections: (**a**) Sputtered and transferred gold on Parylene, (**b**) printed GenesInk, (**c**) transferred silicon die, including printed conductive path, and (**d**) printed contact pad on transferred silicon die.

The cross-sections of the transferred structures are investigated by light microscopy and are depicted in Figure 10. For the transferred metal structures (a,b), the images prove that the conductive paths are embedded into the Parylene, as sketched in Figure 1 (line 6). In contrast to this, the transferred chip is not fully embedded into the Parylene (c). However, the Parylene conformally coats the printed contact pads on the transferred chip (d).

**Figure 11:**
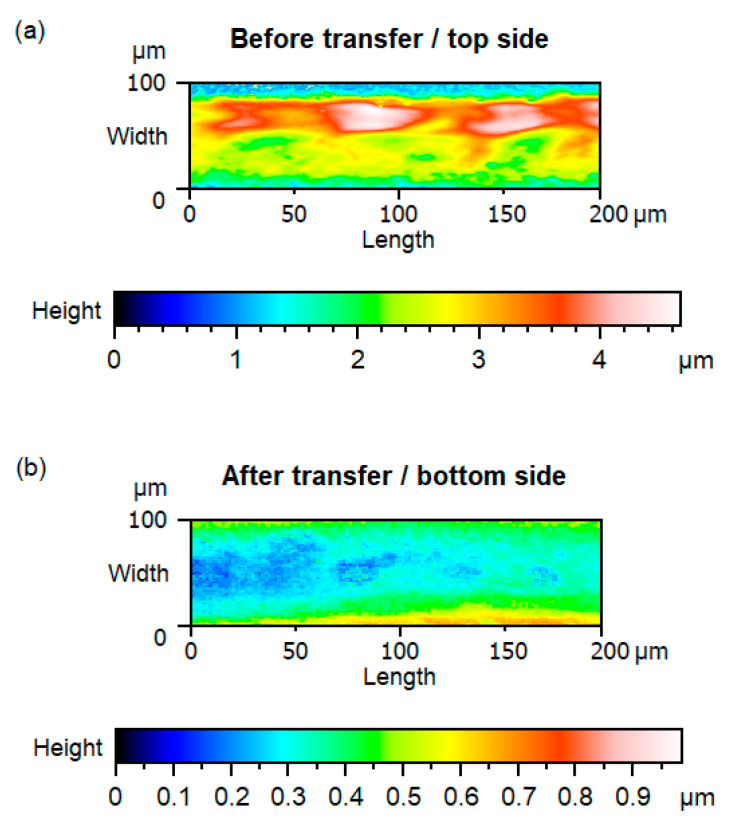
False color images of the sample height, measured by confocal microscopy before (**a**) and after (**b**) the transfer process. For the investigation, a GenesInk-printed conductive path was used.

The results of the roughness measurements of the printed and transferred structure by confocal microscopy are depicted in Figure 11. From the results, it can be concluded that the roughness before the transfer process is much higher than after the transfer process. Note that the electrically accessible side was measured each. Thus, before the transfer, the top side of the printed conductive path with a visible sinter structure was measured, whereas, after the transfer, the bottom side of the printed conductive path was measured. Nevertheless, due to the transfer process, finally, a smooth surface was electrically accessible, even though the original printed structure was rough. Thus, further electrical contact is most likely improved due to the increased surface quality. 

## 4. Discussion

The results successfully demonstrate the feasibility of the transfer of metallic structures, dies, and components as well as whole setups of dies or components with metallic conductive paths from rigid substrates to flexible Parylene substrates. 

Aside from the novelty of the method for the integration itself, this transfer technology overcomes the limitations of ultra-thin, flexible and temperature-sensitive materials that impede the usage of established integration technologies, such as wire bonding, soldering or printing, and dispensing, respectively. Hence, it closes an open research gap for the integration of dies and components on flexible substrates, such as the ultra-thin flexible Parylene PCB [11]. Additionally, the described new transfer process can be an alternative to fan-out wafer packaging [31].

Moreover, the investigated transfer technology enables the merger of conventional wafer processing by microsystem technologies and packaging technologies. By doing so, smaller structure sizes can be realized on flexible substrates, and hence, the next level of miniaturization is enabled. The improved integration of dies, chips and discrete components also enables a smoother fabrication with fewer fabrication steps, particularly considering that the Parylene substrate can also act as an encapsulation and barrier layer. Hence, the presented transfer technology helps to reduce energy consumption and save resources. In summary, the transfer technology enables a unique combination of the advantages of different microsystem technologies and packaging technologies to realize the next generation of ultra-thin flexible electronics. 

Considering the fact that the Parylene coating and sacrificial layer release are the last process steps in the presented technology flow, particularly, fabrication and integration processes with an increased thermal budget are avoided. Hence, the alteration of the Parylene properties is prevented as well [32].

Finally, the described transfer process can be used to increase the adhesion of metallic structures on Parylene as well as to fabricate flexible metallic surfaces of high smoothness in similarity to template-stripped metals described elsewhere [33]. This relates particularly to printed structures, which usually feature a high roughness due to their sinter structure and can be advantageous for improving the electrical contact resistances.

Hence, the presented technology is an advantageous tool for multiple applications.

## 5. Conclusions

A new and efficient process was established for the transfer of sputtered and printed metallic structures to a flexible substrate. The successful transfer was demonstrated for a variety of geometries, thicknesses, metals and metallization technologies. The transferred structures were found to be bendable until a minimal bending diameter of 0.5 cm without any damage and with a reversible change in the electrical resistance, independent of the metal and applied metallization technology. The adhesion of the transferred structures to the Parylene was proven to be excellent and better than for the direct metallization. Finally, cross-sectional images and an investigation of the roughness of the transferred structures reveal more details of the performance of the new process.

Furthermore, the transfer of thinned silicon dies and packaged electrical components was demonstrated successfully using the established process. In doing so, all electrical contacts of the chips are transferred as well. 

Thus, the presented technology closes a gap between the established micro technologies on rigid substrates, such as wafers, and processing on flexible substrates. Hence, the demonstrated technology provides a new and fundamental, paradigm-changing opportunity for the fabrication of flexible electronics by combining the established microsystem technologies, additive manufacturing, pick-and-place, and conventional packaging technologies. 

Additionally, the presented transfer technology can be used to integrate components on the ultra-thin, flexible Parylene PCB [11]. Particularly, this new method overcomes the limitations that conventional packaging technologies, such as wire bonding, soldering or direct printing, feature on ultra-thin, flexible and temperature-sensitive substrates. 

## Figures and Tables

**Table 1:** Diameters of test specimens and related sample strains used for the bending tests.

**Diameter d [cm]**	10	5	3	2	1	0.5	0.3
**Strains ε [%]**	0.01	0.03	0.05	0.07	0.15	0.30	0.50

**Table 2:** Obtained dimensions, electrical conductivities before and after the transfer process, as well as transfer results for the different metals and deposition technologies. The values represent averaged results over three measurements each. The error given for the dimensions relates to the standard deviation.

Material	Deposition Technology	Deposition Parameters	Line Width w [µm]	Line Thickness h [nm]	Electrical Conductivity before Transfer [10^6^ S/m]	Electrical Conductivity after Transfer [10^6^ S/m]	Transfer
Ag (Bando Ink)	Aerosol Jet	1 printing cycle	159 ± 37	2870 ± 1650	1.77	1.90	Partial
		2 printing cycles	228 ± 33	3973 ± 1620	1.48	1.62	Partial
Ag (GenesInk)	Aerosol Jet	1 printing cycle	147 ± 15	690 ± 100	1.48	2.36	Complete
Au	Sputtering	400 s 75 µm mask width	103 ± 7	28 ± 3	8.42	8.44	Complete
		400 s 100 µm mask width	122 ± 4	28 ± 3	10.39	10.46	Complete
		750 s 75 µm mask width	103 ± 9	118 ± 9	5.03	5.08	Complete
		750 s 100 µm mask width	117 ± 5	118 ± 9	6.04	6.08	Complete
Pt	Sputtering	400 s 75 µm mask width	95 ± 1	46 ± 3	0.61	0.63	Complete
		400 s 100 µm mask width	121 ± 5	46 ± 3	0.80	0.82	Complete
		750 s 75 µm mask width	92 ± 4	65 ± 3	0.72	0.56	Complete
		750 s 100 µm mask width	133 ± 5	65 ± 3	0.61	0.63	Complete

**Table 3:** Electrical resistance measurement results for platinum metallized die before and after the transfer process.

Sample Number-	Electrical Resistance before Transfer Process [Ω]	Electrical Resistance after Transfer Process [Ω]
1	5.6	43.8
2	7.8	91.6

**Table 4:** Adhesion of metals on Parylene for direct metallization and metallization by the transfer process.

Metal/ Technology	Direct Metallization	Metallization by Transfer
	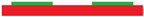	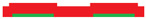
Sputtered Gold	poor	excellent
Sputtered Platinum	good	excellent
Printed Silver (Bando Ink)	fair	good
Printed Silver (GenesInk)	good	excellent

## Data Availability

The data presented in this study are available on request from the corresponding author.

## Appendix B

In contrast to the directly metallized Parylene samples (Figure A2a), transferred metals feature an increased interface area with Parylene (Figure A2b). The process for the fabrication of a transferred metal structure without the increased Parylene interface is depicted in Figure A2c. Similar to the process depicted in Figure 1a, it consists of aerosol jet printing on a PVA-coated silicon wafer using GenesInk S-CS31506. Next, the sample is spin-coated with photoresist OiR906-12 (1000 rpm for 10 s) and subsequently baked at 95 °C for 2 min and hardened overnight in ambient temperatures. To support the photoresist, a Revalpha thermal release tape (Revalpha No. 3195V) was attached, followed by dissolving the PVA in water at ambient temperatures. The sample was subsequently coated with Parylene and lifted by dissolving the photoresist in acetone overnight at ambient conditions.

## References

[1] Yole Developpement SA (2022). Status of MEMS Industry 2022: Market and Technology Report.

[2] Nag A., Mukhopadhyay S.C., Kosel J. (2017). Wearable Flexible Sensors: A Review. IEEE Sens. J..

[3] Unno Y., Affolder A.A., Allport P.P., Bates R., Betancourt C., Bohm J., Brown H., Buttar C., Carter J.R., Casse G. (2011). Development of n-on-p silicon sensors for very high radiation environments. Nucl. Instrum. Methods Phys. Res. A.

[4] Liou J.J., Schwierz F. (2003). RF MOSFET: Recent Advances, Current Status and Future Trends. Solid State Electron..

[5] Costa J., Ivanov T., Carrol M., Hammond J., Glass E., Jorgenson J., Denning D., Kerr D., Reed J., Ren Q. Silicon RFCMOS SOI technology with above-IC MEMS integration for front end wireless applications. Proceedings of the IEEE Bipolar/BiCMOS Circuits and Technology Meeting.

[6] Baum M., Saeidi N., Vogel K., Schroeder T., Selvam K.G.M., Wiemer M., Otto T. An improved design for 2D arrays of capacitive micromachined ultrasound transducers: Modeling, fabrication, and characterization. Proceedings of the IEEE International Ultrasonics Symposium (IUS).

[7] Azevedo R.G., Jones D.G., Jog A.V., Jamshidi B., Myers D.R., Chen L., Fu X., Mehregany M., Wijesundara M.B.J., Pisano A.P. (2007). A SiC MEMS Resonant Strain Sensor for Harsh Environment Applications. IEEE Sens. J..

[8] Choi M.C., Kim Y., Ha C.S. (2008). Polymers for Flexible Displays: From Material Selection to Device Applications. Prog. Polym. Sci..

[9] Chen P.-J., Kuo W.-C., Li W., Yang Y.-J., Tai Y.-C. Q-enhanced fold-and-bond MEMS inductors. Proceedings of the IEEE NEMS.

[10] Mostafalu P., Sonkusale S. (2014). Flexible and transparent gastric battery: Energy harvesting from gastric acid for endoscopy application. Biosens. Bioelectron..

[11] Selbmann F., Roscher F., de Souza Tortato F., Wiemer M., Otto T., Joseph Y. An ultra-thin and highly flexible multilayer Printed Circuit Board based on Parylene. Proceedings of the IEEE Smart Systems Integration (SSI).

[12] Hu Y., Wang S., Zhang H., Wang Y., Hang C., Wen J., Tian Y. (2021). Silver flake/polyanilinie composite ink for electrohydrdynamic printing of flexible heaters. J. Mater. Sci. Mater. Electron..

[13] Wang S., Fen Y., Zhang H., Peng Q., Tian Y. (2021). Highly stable and printable Ag NW/GO/PVP composite ink for flexible electronics. Flex. Print. Electron..

[14] Aqueveque P., Osorio R., Pastene F., Saavedra F., Pino E. Capacitive sensors array for plantar pressure measurement insole fabricated with flexible PCB. Proceedings of the 2018 40th Annual International Conference of the IEEE Engineering in Medicine and Biology Society (EMBC).

[15] Cao S., Pyatt S., Anthony C., Kubba A., Kubba A., Olatunbosun O. (2016). Flexible Bond Wire Capacitive Strain Sensor for Vehicle Tyres. J. Sens..

[16] Segev-Bar M., Haick H. (2013). Flexible Sensors Based on Nanoparticles. ACS Nano.

[17] Chang W.-Y., Fang T.-H., Lin H.-J., Shen Y.-T., Lin Y.-C. (2009). A large area flexible array sensors using screen printing technology. J. Disp. Technol..

[18] Jung M., Kim J., Noh J., Lim N., Lim C., Lee G., Kim J., Kang H., Jung K., Leonard A.D. (2010). All-Printed and Roll-to-Roll-Printable 13.56-MHz-Operated 1-bit RF Tag on Plastic Foils. IEEE Trans. Electron Devices.

[19] Zheng Y., He Z., Gao Y., Liu J. (2013). Direct desktop printed circuits- on-paper flexible electronics. Nat. Sci. Rep..

[20] Krebs F.C. (2009). Fabrication and Processing Of Polymer Solar Cells: A Review Of Printing And Coating Techniques. Sol. Energy Mater. Sol. Cells.

[21] Sandström A., Dam H.F., Krebs F.C., Edman L. (2012). Ambient fabrication of flexible and large-area organic light-emitting devices using slot-die coating. Nat. Commun..

[22] Kim T., Jung Y.H., Chung H.J., Yu K.J., Ahmed N., Corcoran C.J., Park J.S., Jin S.H., Rogers J.A. (2013). Deterministic Assembly of Releasable Single Crystal Silicon-Metal Oxide Field-Effect Devices Formed from Bulk Wafers. Appl. Phys. Lett..

[23] Godlinski D., Zichner R., Zöllmer V., Baumann R.R. (2017). Printing technologies for the manufacturing of passive microwave components: Antennas. IET Microw. Antennas Propag..

[24] Seifert T., Baum M., Roscher F., Wiemer M., Gessner T. (2015). Aerosol Jet Printing of Nano Particle Based Electrical Chip Interconnects. Mater. Today Proc..

[25] Kim M., Shah A., Li C., Mustonen P., Susoma J., Manoocheri F., Riikonen J., Lipsanen H. (2017). Direct transfer of wafer-scale graphene films. 2D Materials.

[26] Kim Y., Kim Y.W., Kim J., Noh M. (2017). A novel fabrication method of Parylene-based microelectrodes utilizing inkjet printing. Sens. Actuators B Chem..

[27] Frühauf J. (2005). Werkstoffe der Mikrotechnik-Lehrbuch für Ingenieure.

[28] Fortin J.B., Lu T.-M. (2004). Chemical Vapor Deposition Polymerization-The Growth and Properties of Parylene Thin Film.

[29] Selbmann F., Baum M., Wiemer M., Gessner T. Deposition of Parylene C and characterization of its hermeticity for the encapsulation of MEMS and medical devices. Proceedings of the IEEE NEMS.

[30] Wuensch D., Purwin L., Buettner L., Martinka R., Schubert I., Junghans R., Baum M., Wiemer M., Otto T. Temporary wafer bonding–key technology for MEMS devices. Proceedings of the IEEE Pan Pacific.

[31] Braun T., Raatz S., Maass U., Van Dijk M., Walter H., Hölck O., Becker K.-F., Töpper M., Aschenbrenner R., Wöhrmann M. Development of a Multi-project Fan-Out Wafer Level Packaging Platform. Proceedings of the IEEE ECTC.

[32] Selbmann F., Scherf C., Langenickel J., Roscher F., Wiemer M., Kuhn H., Joseph Y. (2022). Impact of Non-Accelerated Aging on the Properties of Parylene C. Polymers.

[33] Platypus Technologies LLC (2022). Ultra-Flat Gold Surfaces. https://www.platypustech.com/gold-thin-films/ultra-flat-gold-films.

